# Psychological Issues Among Caregivers of Children with Neurodevelopmental Disorders: A Scoping Review

**DOI:** 10.3390/brainsci16090982

**Published:** 2026-09-16

**Authors:** Gerald Agyapong-Opoku, Ejemai Eboreime, Andrew J. Greenshaw, Belinda Agyapong

**Affiliations:** 1 Department of Psychiatry, University of Alberta, Edmonton, AB T6G 2H5, Canada; 2 Department of Psychiatry, Dalhousie University, Halifax, NS B3H 2E2, Canada; 3 Department of Community Health and Epidemiology, Dalhousie University, Halifax, NS B3H 1V7, Canada

**Keywords:** caregivers, stress, anxiety, depression, intellectual developmental disability, learning disability, mental health, neurodevelopmental disorder, psychological issues

## Abstract

**Background:** Caring for children with neurodevelopmental disorders can be challenging and is normally associated with a heightened risk of psychological challenges among caregivers. **Objective:** This scoping review aims to synthesize the existing literature on the prevalence and correlates of stress, anxiety, and depression among caregivers of children with neurodevelopmental disorders. **Methods:** This scoping review was conducted following the methodological framework outlined by Arksey and O’Malley and the Preferred Reporting Items for Systematic Reviews and Meta-Analyses (PRISMA-ScR) extension for scoping reviews. A comprehensive literature search was conducted in PubMed, PsycINFO, Scopus, Web of Science, and CINAHL. **Results:** A total of 56 studies were included in this review. Factors associated with caregivers’ psychological issues include the age of the child, daily stress from own needs and the child’s care needs, and co-morbidity in the child. Articles utilized different screening tools and diverse diagnostic classifications to determine symptoms of stress, anxiety, and depression, leading to variations in prevalence estimates. The lowest prevalence of stress reported was 7.9%, while the highest reported caregiver moderate-to-severe stress was 94.6%. Similarly, the prevalence of anxiety and depression varied from 6.0% to 85.8% and from 5.2% to 85.1%, respectively. This substantial variability is likely attributable to the heterogeneity of assessment tools and inconsistencies in the use of classificatory terminology, such as “severe,” “clinical range,” and “high”, which may limit the generalizability of the findings. **Conclusions:** Caregivers of children with neurodevelopmental disabilities experience an immense psychological burden. Expanding access to sustainable psychosocial and social protection interventions is a vital step toward improving their well-being. This will indirectly improve the overall outcomes for the children. Government and policymakers should prioritize the implementation of measures such as social support allocated to this cohort to enhance their quality of life and improve their overall well-being.

## 1. Introduction

Neurodevelopmental disorders are a group of conditions characterized by atypical brain development, resulting in impairments in cognitive, behavioral, social, or motor functioning, typically emerging during early developmental periods and often persisting throughout the lifespan [1]. These disorders include conditions such as autism spectrum disorder, Down syndrome, cerebral palsy, and intellectual disability, and they are associated with difficulties in learning and communication [2,3]. Intellectual disability, currently the accepted terminology, was historically referred to as mental retardation and is also considered a neurodevelopmental disorder associated with deficits in both intellectual functioning and adaptive functioning [4].

Caregiving may be a demanding task; however, caring for children with neurodevelopmental disorders presents unique challenges that extend far beyond the demands of typical parenting. Neurodevelopmental disorders, such as intellectual disability, autism spectrum disorder, Down syndrome, and other developmental conditions, are characterized by significant limitations in cognitive functioning and adaptive behaviors that manifest during the developmental period [2,3], affecting about 1% to 3% of the population [4]. Recent advancements in medical care and early intervention services have contributed to the improvement in outcomes for children with intellectual disorders; nevertheless, caregiving responsibilities remain intensive, often requiring continuous emotional, physical, and financial support [5].

Caregiving burden is usually heightened and has been strongly associated with an increased risk of mental health challenges among primary caregivers, including parents, siblings, and relatives. Published literature shows evidence indicating that caregivers of children with intellectual developmental disorders experience elevated levels of stress, anxiety, depression, and reduced overall psychological well-being compared to caregivers of children without developmental issues [6,7]. In addition to experiencing psychological difficulties, they may also experience poorer quality of life, social marginalization, financial difficulties, inability to work, or unemployment, and other work-related problems or challenges based on their life circumstances [8]. All these factors can adversely impact their overall well-being. Moreover, caregivers of children with neurodevelopmental disorders are affected by both psychosocial distress and notable child physical impairments [9,10]. Caregivers of children with autism, in particular, often experience sleep deprivation and persistent sleep disturbances, contributing to serious health outcomes [11]. Factors contributing to this increase have been suggested to include caregiving demands, social isolation, limited access to respite services, lack or discontinuity of therapeutic activities, and financial strain [12,13]. Additionally, societal stigma and a lack of adequate healthcare resources can further exacerbate caregivers’ vulnerability to mental health problems [14].

Given the vital role of caregivers, understanding the prevalence and correlates of mental health challenges among this population is essential for informing policy, service provision, and the design of effective interventions. While several studies have examined the mental health status of caregivers across different regions and populations, findings are dispersed. This Scoping review will provide a valuable methodological framework for synthesizing such heterogeneous evidence, mapping key themes, and identifying disparities in caregiving roles between mothers and fathers, for instance, and research gaps [15].

### Objective

This scoping review aims to systematically map the existing literature on the prevalence and correlates of psychological issues (stress, anxiety, and depression) among caregivers of children with neurodevelopmental disorders, such as intellectual developmental disorders. Specifically, this scoping review will identify the range of reported psychological issues, summarize their prevalence, and highlight contextual factors that may influence caregivers’ mental health. It will also map gaps in the current literature. By collating and analyzing this body of evidence, the review will provide a comprehensive overview of the literature and inform future research, practice, and policy aimed at supporting general caregiver well-being.

## 2. Methods

This scoping review was conducted following the methodological framework outlined by Arksey and O’Malley [15] and reported in accordance with the Preferred Reporting Items for Systematic Reviews and Meta-Analyses extension for scoping reviews (PRISMA-ScR) [16]. This method includes five key stages: identifying the research question, identifying relevant studies, study selection, charting the data, and collating, summarizing, and reporting the results. The PRISMA checklist has been provided as a Supplementary File.

### 2.1. Research Question

The primary research question was: “What is the prevalence and correlates of psychological issues (stress, anxiety, depression) among caregivers of children with neurodevelopmental disorders?”

### 2.2. Eligibility Criteria

The inclusion and exclusion criteria for this scoping review are defined according to the Population Concept Context (PCC) framework, consistent with the methodological guidance from the Joanna Briggs Institute [17,18].

#### 2.2.1. Population

Studies were included if they involved primary caregivers, such as parents, guardians, or family members, of children aged 0–18 years with neurodevelopmental disorders. Neurodevelopmental disorders considered included intellectual disability, autism spectrum disorder, Down syndrome, epilepsy, cerebral palsy, learning disability, global delay and developmental delay.

#### 2.2.2. Concept

The review focused on mental health conditions (stress, anxiety, depression, and. post-traumatic stress disorder). Eligible studies focused on psychological outcomes, specifically psychological distress and mental health conditions, including stress, anxiety, depression, and post-traumatic stress disorder. All peer-reviewed studies were considered, regardless of setting or country. Study designs included quantitative cross-sectional, longitudinal survey, randomized controlled trial and mixed-methods studies that reported prevalence or related outcomes. Systematic reviews and meta-analyses were excluded. Gray literature was excluded to ensure consistency in methodological quality and to focus the synthesis on peer-reviewed evidence that meets established academic standards. Interventional studies were excluded as the aim was not to evaluate intervention effectiveness. Studies were also excluded if they focused solely on children without neurodevelopmental disorders, addressed physical health outcomes without psychological components, or were opinion papers, editorials, or case reports.

#### 2.2.3. Context

There were no restrictions on the geographic location of the included studies. However, due to resource constraints, only peer-reviewed studies published in English were included, but there was no restriction on the publication dates. The included studies were published between 1999 and 2025.

### 2.3. Information Sources and Search Strategy

A comprehensive literature search was conducted in PubMed, PsycINFO, Scopus, Web of Science, and CINAHL (Cumulative Index of Nursing and Allied Health Literature). Search terms combined keywords and Medical Subject Headings (MeSH) related to caregivers, mental health, and neurodevelopmental disorders. The search string included terms such as (Father or mother or caregiver* OR parent* OR guardian* AND Child* intellectual disability OR child* intellectual developmental disability OR child* developmental delay OR child* neurodevelopmental disorder OR Child* Learning disability OR Child* autism OR child* autism spectrum disorder OR Child* Down syndrome OR Child* cerebral palsy OR Adolescen* intellectual disability OR Adolescen* intellectual developmental disability OR Adolescen* developmental delay OR Adolescen* neurodevelopmental disorder OR Adolescen* Learning disability OR Adolescen* autism OR child* autism spectrum disorder OR Adolescen* Down syndrome OR Adolescen* cerebral palsy AND mental health OR depression OR anxiety OR stress OR psychological distress OR mental health issues OR major depressive disorder OR anxiety disorder OR generalized anxiety disorder).

Appendix A gives the electronic search strategy for PubMed. In addition to database searches, reference lists of included studies were screened to identify other relevant articles.

### 2.4. Study Selection

**Figure 1:**
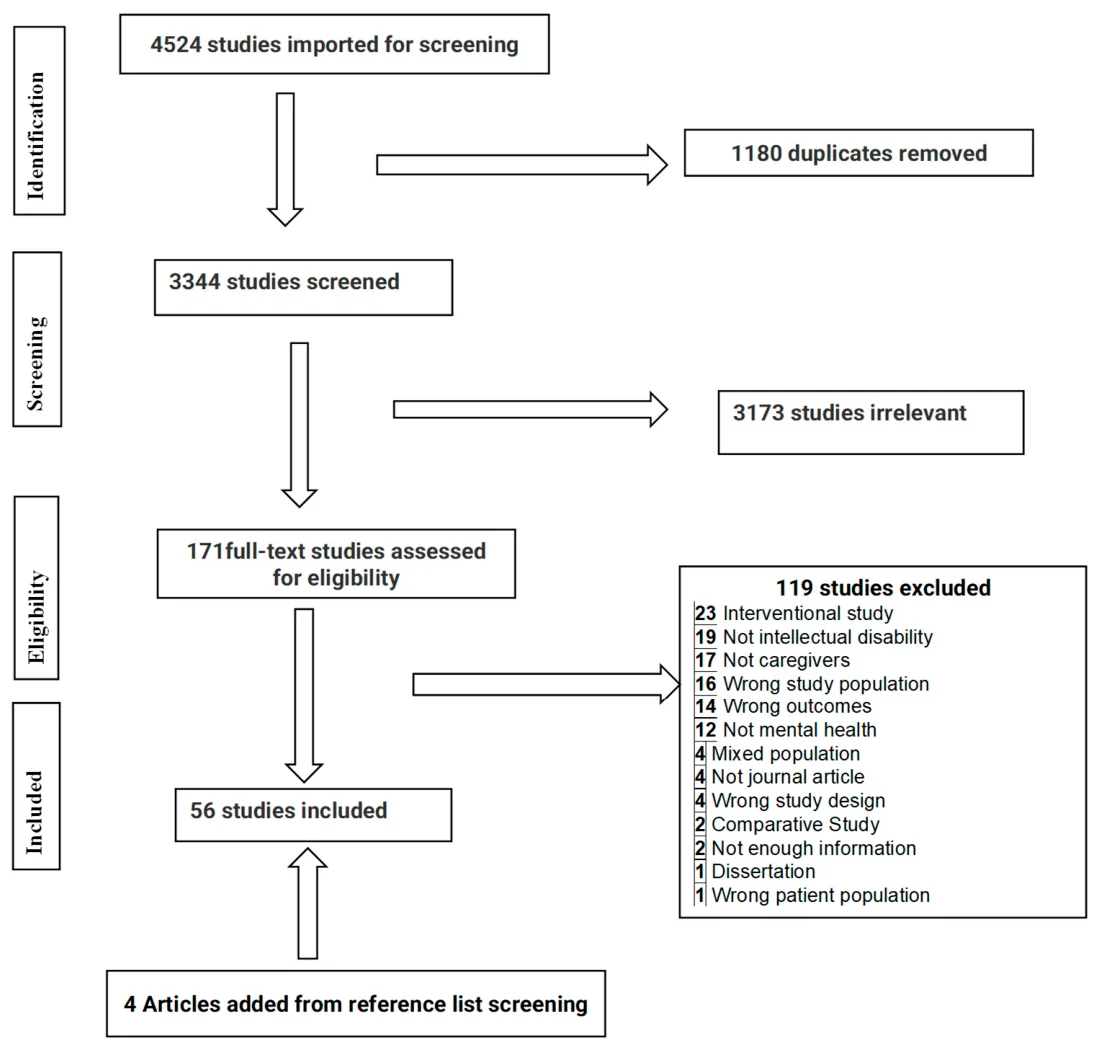
PRISMA diagram outlining the selection process.

All retrieved articles were imported into Covidence web-based software platform, Melbourne, Australia [19], and duplicates were automatically removed. Two independent reviewers screened the titles and abstracts against the eligibility criteria, followed by full-text screening of articles deemed potentially relevant. Discrepancies between reviewers were resolved through discussion or consultation with a third reviewer. The PRISMA flow diagram, Figure 1 below, summarizes this information in detail.

### 2.5. Data Extraction and Charting

A standardized data extraction form, developed by researchers, was used to chart key study characteristics, including author, year of publication, country, study design, sample size, caregiver demographics, type of neurodevelopmental disorder, and mental health outcomes assessed, measurement tools, correlates, and prevalence estimates. Extracted data were cross-checked by a second reviewer to ensure accuracy.

### 2.6. Data Synthesis

Descriptive synthesis, combined with thematic induction, with a clear visualization approach, was utilized for the analysis, including tables and diagrams. Specifically, themes were inductively derived based on key dimensions such as mental health of caregivers by continent, correlates, prevalence patterns, and categories of associated factors. Extracted data were summarized into tables, and where possible, prevalence range estimates for different mental health outcomes were recorded. Patterns and trends across studies were identified, considering factors like caregiver demographics, regional differences, and child neurodevelopmental conditions.

## 3. Results

### 3.1. Overview of Included Studies

The search strategy yielded 4524 citations. Using Covidence software [19], 1179 duplicates were automatically removed, and one additional duplicate was removed manually. A total of 3344 studies remained for full-text screening, of which 171 proceeded to full-text review. A total of 119 studies were excluded, resulting in 52 studies. A review of the reference list from other studies was also conducted, resulting in four additional eligible articles. Thus, a total of 56 studies were included in this review, as illustrated in Figure 1. Across these 56 studies, there were 22,540 caregivers, the majority consisting of parents (mothers or fathers) or other relatives, with only one study that included friends and service providers. The sample sizes in the included articles (Table 1) ranged from 21 to 9467 caregivers, with participants’ ages spanning 17 to 85 years. The studies were published between 1999 and 2025. An equal number of studies (N) (n = 28) were published in the periods up to 2019 and from 2020 to 2025. Between 2019 and 2021, corresponding to the early phase of the COVID-19 pandemic, a total of 12 studies were published. As presented in Table 1, thirty-three studies focused on a single neurodevelopmental condition, with three studies [20,21,22] stating “neurodevelopmental disorders”. Eight studies examined two conditions (learning disability and epilepsy; autism and intellectual disability; global delay and developmental delay; intellectual and developmental disabilities), and 15 studies included three or more neurodevelopmental conditions. Distribution of studies by continent: most studies were conducted in Asia (43%), followed by Europe (27%), North America (16%), Africa (9%), and Oceania (3%). Notably, no studies were conducted in South America, and one study (2%) [13] was conducted across multiple locations (USA, Spain, and Italy).

**Table 1:** (A) Mental health burden among caregivers (2020–2025). (B) Mental health burden among caregivers (1999–2019).

**Author/(Year)**	**Country**	**Study Design**	**Sample Size**	**Scales Utilized**	**Caregiver** **Demographics**	**Child’s Disorder**	**Caregivers Mental Health Burden**	**Main Findings**
**(A)**								
Ramachandran, 2020 [23]	India	Cross-sectional study	101	Kingston Caregiver Stress Scale	Family members ≥35 years = 67 ≤36 years = 34	Autistic Spectrum Disorder, Intellectual Disability	Stress	64.3% of the caregivers had a severe level of stress, 21.7% of the caregivers had a moderate level of stress, and 13.8% of the caregivers had mild stress. Caregiver level of stress was associated with economic status, type of family, and gender of caregiver.
Chen, 2020 [24]	China	Questionnaire/survey	1450	General Health Questionnaire (GHQ), Perceived Social Support, Parenting Stress Index, and Neuroticism, Extraversion, Openness Five Factor Inventory	Parents age range 40.76	Autism spectrum disorder, Intellectual disability	Anxiety, depression, marital, and sleep problems	27% had poor mental health. Parents of children with autism spectrum disorder were more likely to have mental health problems compared to parents whose children had an intellectual disability or a visual or hearing impairment.
Jijon, 2020 [25]	U. K	Survey	174	Parenting Stress Index, Family Support Scale	Parents parents age M = 42.3, SD = 4.11	Developmental Coordination Disorder	Stress	Two-thirds of parents reported clinically significant levels of stress. Higher levels of behavioral problems and child’s lower motor proficiency were associated with increased parenting stress.
Dabilgou, 2021 [26]	Burkina Faso	Cross-sectional study	100	The State-Trait Anxiety Inventory scale and Beck Depression Inventory scale were used to assess anxiety and depression.	Primary caregivers 37.75 ± 10.69 years	Generalized epilepsy	Anxiety and depression, many parents experienced fatigue, intimacy problems with their partner, deterioration of mental health, and work restrictions resulting from caregiving responsibilities	Anxiety was observed in 56%, depression in 27%, and both anxiety and depression in 23% of caregivers.
Suarez-Balcazar, 2021 [27]	United States	Cross-sectional study	37	Medical Outcomes Study Questionnaire Short Form 36 Health Survey (SF-36). Caregiver mental health was measured by the 20-item Center for Epidemiological Studies Depression Scale (CES-D)	Mothers 43.9 (6.9)	Intellectual and developmental disabilities	Depression	Perceived social support, annual family income, food security, and receipt of financial benefits were correlated with fewer depressive symptoms. Annual family income was also significantly correlated with perceived general health. Moderately high overall depressive symptoms (MCES-D = 12.2), with 36% at risk of having depression (CES-D scores ≥ 16). More than one-third of the caregivers were at risk of depression.
Sharma, 2021 [28]	India	Cross-sectional study	197	Hospital Anxiety and Depression Scale (HADS)	Parents ≤30 and >40	Intellectual disability	Depression and anxiety	94% of mothers and 66.7% of fathers were found to have either anxiety or depressive symptoms, or both. Among mothers, 91.8% had scores suggestive of anxiety, 66.3% for depression, and 64.3% for both anxiety and depression. Among fathers, 57.6% had scores suggestive of anxiety, 35.4% for depression, and 26.3% for both. Caregivers’ depression was associated with the severity of the child’s intellectual disability. Diagnosis of Down syndrome and lack of family support. Fathers’ anxiety and depression scores were associated with the age of the father and the child’s medical co-morbidities.
Fatima, 2021 [10]	India	Cross-sectional study	117	Patient Health Questionnaire-9 (PHQ-9) and Generalized Anxiety Disorder-7 (GAD-7) questionnaire	Parents Most were 30 years and above	Neuro-developmental disorders, epilepsy, intellectual disability, cerebral palsy	Anxiety and depression	37.5% depression, 43.3% anxiety, 30% both. Factors associated with a diagnosis of both depression and anxiety include family history of psychiatric disorder, male gender of the child, co-morbidity in children, and child diagnosis.
Maridal, 2021 [20]	Nepal	Semi-structured interview	63	General Health Questionnaire-12, Gross Motor Function Classification System	Primary caregiver, mostly mothers Age in years (19–68). 31.51 (8.53) SD	Neurodevelopmental disorders	Anxiety, depression, and stress	Almost half (46%) scored six or higher, indicating a high level of distress. A majority of the caregivers reported that caring for their disabled child had a negative effect on the caregiver’s economy (70%), physical health (65%), social life (64%), and dreams and expectations for the future (81%). There was a significant relationship between the caregiver’s psychological distress (GHQ-12) and the degree of disability in the child.
Kelada, 2021 [29]	India	Survey	205	Hospital Anxiety and Depression Scale, Brief Illness Perception Questionnaire and Coping Health Inventory for Parents	Parents	Neurological disorder	Depression and anxiety	As these illness perceptions increased, maintaining social support decreased, and symptoms of depression increased. Mild to severe symptoms of anxiety (41.0%) and depression (39.5%).
Ibrahimagić, 2022 [30]	Bosnia	Survey	80	Authors Research Questionnaire	Parents Age-NR	Autistic Spectrum Disorder	Stress and psychological distress	Parents of a child with ASD often have higher levels of stress and psychological distress. Frequent stress, especially for these variables: “Caring for the child’s future”; “Difficulties of the child with speech, language, and communication”; “Difficulties of the child in establishing contact with peers and other persons” and “Inappropriate behavior of the child” (anger, aggression, stereotypical behaviors).
Miniarikova, 2022 [31]	France	Cross-sectional study	134 parents	The Hospital Anxiety and Depression Scale (HADS)	Mothers and fathers The mean age was 41.1 years (±6.8) for the mothers and 44.2 years (±7.8) for the fathers.	Autism spectrum disorder	Anxiety and depression	39.4% (n = 37) showed combined anxiety + depression. More mothers with combined anxiety + depression reported worsening of their child’s challenging behaviors than those without.
Odedra, 2022 [32]	India	Cross-sectional study	220	Patient Health Questionnaire (PHQ), Hamilton Depression Rating Scale (HAM-D)	Majority female caregivers 18–60	Intellectually differently abled	Depression	Prevalence of major depressive disorder in caregivers as per DSM-5 was 56 (25.45%). A total of 51 (23.18%) caregivers had severe burden, 100 (45.45%) moderate to severe burden, 61 (27.72%) mild to moderate burden, and 8 (3.63%) caregivers had little or no burden. The association between intellectually differently abled persons with psychiatric and non-psychiatric co-morbidities and caregivers’ depression was statistically significant.
Johny, 2022 [33]	India	A Case–control study	204	General Health Questionnaire- 28	Mother Mean age of the mothers recruited in each group is as follows: 36.15 ± 6.13 years (ASD group), 33.77 ± 8.94 years (ID group), 35.39 ± 7.07 years (TD group).	Autism spectrum disorders and intellectual disability	Psychological distress, anxiety/insomnia, social dysfunction, depression	Higher levels of prevalence rates of psychological distress for mothers with ASD than ID children. Mothers of neurodevelopmental disorder: 14.28%), anxiety/insomnia (21.4%), social dysfunction (14.28%), depression (17.87%), and overall psychological distress (17.87%). Prevalence for maternal distress among the children with ASD group: somatic symptoms (15.78%), anxiety/insomnia (42%), social dysfunction (26.3%), depression (15.78%), and overall psychological distress (36.84%). Prevalence of maternal distress among children with ID group: somatic symptoms (11.11%), anxiety/insomnia (33.33%), social dysfunction (22.22%), depression (22.22%), and overall psychological distress (22.22%).
Mazzoni, 2022 [13]	Italy, Spain, USA	Cross-cultural study Mixed method	1494	GHQ	Parents Italy 42.86 (7.39) Spain 41.70 (6.29) USA 42.32 (5.92)	ASD, deficit and hyperactivity disorder, language disorder, and learning disabilities	Stress	Inadequacy and sense of helplessness. (12.09%). Quantitative results showed that increased parental stress may have contributed to a worsening in parental psychological distress, regardless of culture, and an indirect effect of child externalizing behaviors on parents’ psychological distress via parental stress. Qualitative analyses highlighted that the lack, or discontinuity, of therapeutic activities may have been one of the key contributors to parenting burden during the COVID-19 pandemic. Stress and negative feelings (n = 20; 21.98%).
Lim, 2022 [7]	Singapore	Cross-sectional survey	107	Depression, Anxiety, and Stress Scales (DASS-21) Connor-Davidson Resilience Scale 25-item	Mean age of 40.5 years (SD = 6.0)	Developmental disabilities: autism spectrum disorder, global developmental delay/intellectual disability	Depression, anxiety, and stress	A significant proportion (73.5%) felt stressed, with 68.2% feeling that their stress levels were higher compared to before the COVID-19 pandemic. On the DASS-21, 52.3% of respondents had a positive screen for depression, 52.3% for anxiety, and 41.1% screened positive for stress. Of those who screened positive for any of these sub-groups, 70.4 to 75.0% were rated in the moderate or more severe category. The most significant self-rated contributors to stress were “school closing and having to manage my child at home for the full day”, and “closure of therapy/intervention services makes me worried about disrupting therapy”.
Alsaad, 2023 [34]	Saudi Arabia	Cross-sectional study	416	Patient Health Questionnaire-9 (PHQ-9) and Generalized Anxiety Disorder-7 (GAD-7)	Parents Parents’ ages ranged from 20 to more than 60 years, with a mean age of 29.2 ± 13.9 years	Neurodevelopmental disorders including specific learning disorders, autism spectrum disorder, intellectual disability	Depression and anxiety	85.1% of parents had depression, and 85.8% had anxiety. Mothers and fathers had similar rates of depression and anxiety.
Mbatha, 2023 [22]	South Africa	Cross-sectional study	500	Ten items of the Parental Stress Scale (PSS)	Parents: mother AND caregivers 19–65, mean of 33.9 (±7.8) years	Neurodevelopmental disorder	Stress, distress	The majority (52.2%) of the participants reported very high clinically significant stress levels (≥85%ile). The four factors that independently and significantly predicted high parental stress were the advanced age of mothers and caregivers, caring for a child with multiple diagnoses, non-school of the child, and frequent hospital visits.
Domaradzki 2024 [35]	Poland	Cross-sectional survey	32	Validated questionnaire (5-Likert scale)	Mean age: 37.7; range: (27–49)	Williams syndrome	Anxiety, depression	Lack of access to rehabilitation, problems with daily activity, lack of time for themselves, work restrictions, fatigue, intimacy problems, deterioration of mental health and problems with sleeping. Also, deterioration of physical health, and weight problems. Anxiety and fear (49.9%), sadness and depression (40.6%), mood swings (46.8%).
Alghamdi, 2024 [36]	Saudi Arabia	Cross-sectional design	111	Patient-Reported Outcomes Measurement Information System-Profile 29	Caregivers, the majority of mothers (65.8%) The mean age was around 40 years old	Developmental disabilities	Anxiety, depression, fatigue, sleep disturbance, and pain interference	12.3% of the variance in the physical function domain, 13.9% in the anxiety domain, 24.7% in the ability to engage in social activities and roles, and 11.4% in the pain interference domain. Caregivers reported higher levels of anxiety, depression, fatigue, sleep disturbance, pain interference, and lower levels of physical function and social participation compared to the general population.
Waqar, 2024 [37]	Islamabad	Cross-sectional study	200	Perceived Stress Scale (PSS-10)	Mothers 20–57 years Mean age = 30.85 years	Down syndrome, autism spectrum disorder (ASD), cerebral palsy (CP), developmental disorders	Stress	The mean perceived stress score (PSS-10) was 31.94. There were significant differences in stress levels between joint and nuclear family caregivers, and those with daughters versus sons with disabilities. Caregiver and child age, time since diagnosis, and the number of children positively predicted, while the income and education of the caregiver negatively predicted perceived stress.
Finless, 2024 [38]	Canada	Survey	100	Screen for adult anxiety-related disorders (SCAARED), PTSD checklist (PCL-5), Parenting trauma checklist (PTC),	Parents/grandparent Mean Age 41.70, 7.22 (SD)	Deletion syndrome	Anxiety and depression traumatic experiences	Depression (39%), anxiety (25%), and post-traumatic stress disorder (PTSD) symptoms (30%).
Pahwa, 2024 [39]	India	Cross-sectional study	100	Hamilton Depression Rating Scale (HDRS) and Hamilton Anxiety Rating Scale (HAM-A).	Parents mean age 32 years 5 months (SD 5 years)	Epilepsy	Anxiety, stress, and depression	26% of mothers reported very high stress levels. The parents of children with communication impairment, the parents of children with moderate or severe pain, and the parents of children with an intellectual impairment had higher odds for very high stress.
Balachandran, 2024 [21]	India	Quantitative research design	150	The Kingston Caregiver Stress Scale	Parents Mean age ≤ 40	Neurodevelopmental disorders	Stress	94.6% experienced moderate–severe stress. Significant negative relationship between self-reported measures of caregiver stress and perceived immune status.
Blanchet et al. 2025 [40]	Florida	Randomized controlled trial (RCT)	150	The Depression, Anxiety, & Stress Scale (DASS-21	Primary caregivers 34.49 SD = 6.32	Developmental delay	Depression, anxiety, & stress	Depression—caregivers falling in the mild to moderate (15.3%) or severe to extremely severe (7.4%) anxiety subscale, mild to moderate (12.6%) or severe to extremely severe (7.2%) stress subscale, mild to moderate (13.4%) or severe to extremely severe (8.1%). Bidirectional associations between child internalizing behavior problems and caregiver depressive symptoms.
Chawki, 2025 [41]	Frances	Cross-sectional study	180	Hospital Anxiety and Depression Scale	Mothers and fathers mean age of participating mothers was 45 years	Developmental coordination disorder	Anxiety, depression, and the quality of life of parents	Parents of children with developmental coordination disorder reported significantly higher anxiety and depression levels. Anxiety scores, 46% of mothers scored in the clinical range (HADS score ≥ 11) and 28.6% in the subclinical range (score 8–10). For depressive symptoms, 5.2% of mothers scored in the clinical range and 19.8% in the subclinical range. Key predictors of parental anxiety included the severity of the child’s behavioral difficulties, expressive suppression, and parental stress. Depression was predicted by cognitive reappraisal and social support, while quality of life was related with child’s behavioral severity and parental stress.
Pardo-Salamanca et al. 2025 [42]	Spain	Survey— self-report questionnaire	120	Parenting Stress Index – Short Form	Mothers Age ranges from 35 to 55 years (M = 42.64; SD = 5.00)	Autism (ASD)	Stress	Multiple regressions showed that confidant support was a significant predictor of parenting stress.
Correale, 2025 [43]	Italy	Cross-sectional study	149	Parenting Stress Index—Short Form (PSI-SF)	Caregivers mostly mothers Mean = 47.7 (SD = 7.94) Range = 23–67years	Epilepsy	Stress	Cognitive and behavioral factors are the key predictors to caregiver stress. Parents of children receiving polytherapy or with drug-resistant epilepsy reported higher levels of dysfunctional parent–child interaction. Lower educational attainment was also linked to greater stress.
Kumari, 2025 [44]	New Delhi	Cross-sectional study	170	Parental Stress Scale (PSS)	Mothers 21 years to 55 years	Intellectual disability	Stress	75.0%, 72.0%, and 53.3% of study subjects experienced high stress who had monthly per capita income of Rs 2000–5000, Rs. 5000–10,000, and Rs. > 100,000, respectively. The association between PSS score and monthly per capita income was found to be highly significant.
**(B)**								
Ong, 1999 [45]	Malaysia	Survey	75	Parenting Stress Index, Wechsler Intelligence Scale for Children	Mothers 36.3 (6.1)	Mental retardation	Stress	Chinese ethnicity (*p* < 0.01) and maternal unemployment (*p* < 0.01) were significant predictors of stress.
Aslam, 2002 [46]	Pakistan	Cross-sectional study	96	Parenting Stress Scale (PSS) and Beck Depression Inventory	Mother, father, and grandparents/siblings	Autism spectrum disorder	Stress and depression	Overall, the mean stress score was 52.3 ± 7.3, ranging from 36.0 to 70.0, and the depression score was 62.5%, where 25% mildly, 11.5%borderline/clinical,16.7% moderately and 9.4% were severely depressed. Mothers, 76 (79.2%), reported more pronounced level of stress as compared to fathers.
Wang, 2004 [47]	Taiwan	Survey	63	Chinese version of the Parenting Stress Index	Parents: fathers and mothers—Fathers: 36.4 ± 5.9. Mothers: 34.1 ± 5.5	Cerebral palsy	Stress	The PSI total scale was significantly higher than that for parents in the comparison group. The PSI total scale score was significantly associated with both the child’s severity of motor disability and age of commencing rehabilitation. Parents of children with developmental disabilities reported greater stress. About half (48%) of parents of children with cerebral palsy responded with high total scale scores, compared with only 25% of parents in the comparison group.
Majumdar, 2005 [48]	India	Mixed method	180	Family Interview for Stress and Coping (FISC) in Mental Retardation, the Hamilton Anxiety Rating Scale	Parents The mean age of fathers in group B was 38.27 years. The mean age of mothers in group C (27.9 years; SD ± 1.07) was comparatively lower	Mentally retarded	Stress and anxiety	Parents with profoundly to moderately mentally retarded children had a significantly higher frequency of stressors and level of anxiety. A positive correlation was found between the level of anxiety and stressors.
Abasiubong, 2006 [49]	Nigeria	Structured questionnaire (survey)	106	General Health Questionnaire	Parent The mean age of the subjects was 40+/−6.6 years	Learning disability	Anxiety, depression, stress	Anxiety (25.5%) and depression (10.4%). Marital difficulties, and higher GHQ scores in mothers of children with learning disabilities.
Khamis, 2007 [50]	United Emirates	Survey	225	Questionnaire on Resources and Stress Short-Form, psychological distress. The psychiatric symptom index, the family environment scale	Fathers/Mothers 21–85	Mental retardation	Stress	Child’s age affected both stress and distress; the severity of the disability affected distress. Fathers’ work appeared to be a significant predictor of parental stress. Lower socioeconomic level was associated with greater symptom rates of cognitive disturbance, depression, anxiety, and despair among parents.
Plant, 2007 [51]	Australia	Survey	105	7-point Likert scale	Parents mothers/fathers = 25–50. mothers mean age = 35.24. Fathers mean age = 38.31	Developmental disability: autism spectrum disorder (23.8%), Down’s syndrome (23.8%), chromosomal abnormality other than Down’s syndrome (8.6%), and cerebral palsy (6.7%)	Stress	Predictors include: difficulty of care-giving tasks, difficult child behavior during care-giving tasks, and level of child disability.
Cushner-Weinstein, 2008 [52]	USA	Cross-sectional study	65	Parenting Stress Index, Child Depression Inventory, and behavior and demographic forms	Parents Age—NR	Learning disability; epilepsy	Stress	Parenting stress related to learning disorders and seizure-related factors. High levels of stress among parents (45%). Seizure-related factors of polytherapy, duration, and age at onset were correlated with depression.
Webster, 2008 [53]	Canada	Survey	65	Parenting Stress Index. Child Health Questionnaire	Parents Age—NR	Developmental delay, poor psychosocial health was a common comorbidity	Stress	More than 40% of parents had a Parenting Stress Index above the 85th percentile (clinically significant stress). High levels of parenting stress were best predicted by a child’s Child Health Questionnaire psychosocial health score.
Gallagher, 2008 [54]	Ireland	Cross-sectional study	61	Hospital Depression and Anxiety Scale,	Parents (mean age/SD) 42.8 (5.78)	Intellectual disabilities	Depression and anxiety	The strongest predictor of psychological morbidity was the caregiver burden. Depression score ≥ (8%) 63%; anxiety score ≥ (8%) 24 (75%) among parents of children with intellectual disabilities. The majority met the criteria for possible clinical depression and/or anxiety.
Glenn, 2009 [55]	England	Randomized controlled study	80	Parenting Stress Index	Mothers Maternal mean age was 30.9 years (SD 2.0, range 17–44 years)	Cerebral palsy	Stress	43% had total PSI scores above the threshold for clinical assessment. Factors most strongly related to parenting stress levels were high family needs, low family adaptability, and cognitive impairment in the child. A total of 40% for the child domain and 30% of the parents’ domain were above the threshold and advised to seek clinical assessment.
Lach, 2009 [9]	Canada	Longitudinal survey	9467 caregivers. 2645—12.30% (n ¼ 1164) cared for a child with a neurodevelopmental disorder, and 15.64% (n ¼ 1481) cared for a child with externalizing behavior problems. A total of 4.37% (n ¼ 441) were caring for a child with both a neurodevelopmental disorder and externalizing behavior problems (Both), 7.92% (n ¼ 750) had a child with a neurodevelopmental disorder only (Neuro Only), and 11.27% (n ¼ 1067) had a child with externalizing behavior problems only	12-item general functioning subscale, Center for Epidemiological Studies’ Depression scale, Social Provisions Scale	Parents of children with both neurodevelopmental disorders and externalizing behavior problems −34.98 years (SD = 4.87) Neurodevelopmental disorders only 35.32 years (SD = 5.16)	Autism spectrum disorder neurodevelopmental disorders: cerebral palsy, epilepsy, mental handicap, and learning disability	Depression Significant burden on their psychological health	Depression—minimal symptoms: 68.22%. Parents of children with Both Neurodevelopmental disorders and Externalizing behavior problems. A total of 85.54% parents of children with neurodevelopmental disorders. Somewhat elevated Depression symptoms: 23.51%. Parents of children with Both Neurodevelopmental disorders and Externalizing behavior problems. 10.73% Parents of children with Neurodevelopmental disorders. Very elevated Depression symptoms, 8.27%. Parents of children with Both Neurodevelopmental disorders and Externalizing behavior problems. A total of 3.73% parents of children with Neurodevelopmental disorders.
Norizan, 2010 [56]	Malaysia	Cross-sectional study	147	Parental Stress Scale (PSS)	Mothers—their ages ranged from 23 to 59 years, with an average age of 43.1 years.	Down syndrome	Stress	The mean parenting stress score of these mothers was 37.6 (SD = 8.1) and their parenting stress scores were normally distributed with minimum and maximum values of 21 and 58, respectively. The behavior problem was significantly associated with parenting stress. Parenting Stress was significantly higher among mothers of children with behavioral problems compared to those without such problems.
Mbugua, 2011 [57]	Kenya	Cross-sectional study	114	Demographic questionnaire and the Beck depression inventory	Parents: 21–70 years	Intellectual disability	Depression	Seventy-nine percent (79%) of the caregivers were at risk of clinical depression. Minimum depression 21.1%, mild depression: 26%, moderate depression: 21%, severe depression: 31.6%. Sociodemographic factors associated with the risk of depression included gender, unemployment, primary education, married status, and the age of the caregiver.
Parkes 2011 [58]	Europe (9 Regions)	Cross-sectional study	785	Parenting Stress Index Short Form	Parent; majority mothers. Age: NR	Cerebral palsy	Stress	Having a child with intellectual impairment was associated with an increased risk for very high parenting stress (OR = 1.8). Parents of children with communication impairment, an intellectual impairment, or with moderate or severe pain had higher odds for very high stress. Prevalence for very high stress was 26%.
Tervo, 2012 [59]	USA	Cross-sectional study	201	Parenting Stress Index scale	Parents mean age 39	Global delay Developmental Delay	Stress	Forty-two percent of parents described clinically significant parenting stress. Parental stress increased with higher gross motor development and decreased as social and fine-motor ratios increased. Furthermore, stress increased when parents reported higher levels on the Emotionally Reactive and Withdrawn scale scores and when parents reported Pervasive Developmental and Oppositional Defiant Problems.
Chiu, 2013 [60]	China	Survey	211	The Loss of Face Scale, General Health Questionnaire, and State-Trait Anxiety Inventory (STAI)	Family members, caregivers, friends, and even service providers age range—NR	Intellectual disability	Anxiety, depression, stress	60.6% severe mental disturbance, which required further medical attention. Mental health is related to the affective dimension of affiliated stigma, loss of face, and anxiety level.
Gallagher, 2014 [61]	Ireland	Cross-sectional study	627	Center for Epidemiological Depression Scale	Parents 39.8 (5.94 SD)	Developmental disability	Depression	Having a child with disabilities was associated with a higher risk of depression compared to parents of typically developing children.
Valicenti, McDermott, 2015 [62]	USA	Cross-sectional study	100	The interview included Parenting Stress Index-Short Form, Gastrointestinal Questionnaire, Child Sleep Habits Questionnaire, and Aberrant Behavior Checklist	Parents ASD: 29 + 8 years. DD: 29 + 6 years	Autism and other developmental disabilities and cerebral palsy, intellectual disability/global developmental delays	Stress	Parental stress was significantly higher for the autism group and for non-Hispanic and US-born mothers. In both study groups, parental stress was related to child irritability. Parental stress was also related to gastrointestinal problems in the autism group and sleep difficulties in the developmental disabilities group. In an ethnically diverse population, at least 45% of families of children with autism spectrum disorder reported significant parental stress compared to 22% of families with DD.
Giallo, 2015 [63]	Australia	Survey	315	Depression Anxiety Stress Scale	Fathers 57.9 (SD 28.5, range 0–114)	Intellectual disabilities	Depression and stress	Strongest predictors of fathers’ mental health difficulties were children’s behavior problems, daily stress from own needs and children’s care needs, and low parenting satisfaction. Symptoms of depression (7.9%), anxiety (6.0%) and stress (7.9%) in the Severe to Extremely Severe ranges. Reported significantly more symptoms of depression and stress than the Australian normative data, approximately 6–8% reporting symptoms in the severe to extremely severe range.
Al-Farsi, 2016 [64]	Oman	Case–control study	21	Depression, Anxiety, and Stress Scale 21	Family—fathers, mothers The mean age (SD) of all parents was 39.7 (4.2) years.	Autism spectrum disorder, intellectual disability	Stress, anxiety, and depression	45.9% of caregivers reported having stress and anxiety, 48.6% reported having depression. Mothers have increased psychological morbidity compared to fathers. Psychological morbidity associated with a child’s disability.
Masulani-Mwale, 2018 [65]	Malawi	Cross-sectional study	170	Self-Reporting Questionnaire (SRQ)	Parents The mean age of the participants was 34.4 years (SD: 9.5).	Intellectual disabilities	Stress	70/170 (41.2%) of parents of children with intellectual disabilities reported psychological distress. Increased perceived burden of care (*p* = 0.05), and having no sources for psychological support (*p* < 0.05) significantly predicted psychological distress among the parents of children with disabilities.
Durukan, 2018 [66]	Turkey	Survey	114	Toronto Alexithymia Scale (TAS-20), Beck Depression Inventory, and State–Trait Anxiety Inventory	Parents Maternal age (years)/SD = 33.9 ± 4.5 Paternal age 36.1 ± 3.9	Pervasive developmental disorders	Anxiety and depression, alexithymia	Alexithymia symptoms were higher in parents of children with autism disorder. Parental depression and state anxiety scores were increased as the child’s symptom severity increased.
Tak, 2018 [67]	India	Survey	60	Beck Depression Inventory and Hamilton Anxiety Rating Scale, Brief Psychiatric Rating Scale	Parents The mean age of the parents was 26.34 years. Most of the parents are between 20 and 30 years old	Intellectual/neurodevelopmental disability	Depression and anxiety	The prevalence of depressive disorder was 28.33%, anxiety disorder was 18.33%, and other psychiatric disorders was 8.33%.
Picardi, 2018 [68]	Italy	Cross-sectional study	644 mothers and 539 fathers	GHQ-12.	Parents: mostly mothers, Mothers’ age: 42.5 ± 6.0. Father’s age: 45.8 ± 6.8	Autism spectrum disorder	Depression, anxiety, and stress	The parents of children with ASD reported higher objective and subjective burden, more frequent psychological distress, and lower social support. Mothers reported greater subjective burden than fathers. Significant depressive and anxiety symptoms were found in a high proportion of parents of children with ASD, as 113 (33.0%) of 342 mothers and 85 (29.9%) of 284 fathers scored above the threshold for probable psychiatric cases on the GHQ-12.
Puka, 2019 [69]	Canada	Cohort study	356	Center for Epidemiological Studies Depression Scale	Mothers 38.1 (SD = 6.2). 4.9.1 (SD = 5.4)	Epilepsy	Depression	57% scored in the at-risk range for major depressive disorder: “Low-Stable” (29% of mothers), “Intermediate-Stable” (46%), “High-Stable” (20%), and “High-Decreasing” (5%). Significant factors included the type of seizures, the child cognitive comorbidity, maternal age, and maternal education.
Cleaton, 2019 [70]	United Kingdom	Mixed-methods study	186 Valid responses	Family QOL Scale and The 12-Item Short Form Health Survey	Parents Average age: 42.8	Developmental coordination disorder	Stress	The lack of support by medical and educational professionals was cited as a major source of stress. Parental mental health was also negatively affected.
Gugala, 2019 [71]	Poland	Survey	301	Hospital Anxiety and Depression Scale, Gross Motor Function Classification System for Cerebral Palsy, Sense of Coherence Scale, and Berlin Support Social Scales	Parents 40 (35–46, 22–69)	Cerebral palsy	Depression and anxiety	Parents of children with cerebral palsy (CP) showed significantly higher anxiety and depression compared to parents of typically developing children (anxiety: 8.1 vs. 4.7; depression: 6.8 vs. 3.7). Higher anxiety and depression were linked to factors such as low social support, poor parental health, financial difficulties, challenging living conditions, low sense of coherence, loneliness, the parent’s gender, and the child’s intellectual disability.

NR: not recorded ASD: autism spectrum disorder SD: standard deviation CP: cerebral palsy.

### 3.2. Assessment Scales

Diverse measurement scales were used in the assessment of stress, anxiety, and depression. For depression: Beck’s Depression Inventory, Patient Health Questionnaire (PHQ), Hamilton Depression Rating Scale (HAM-D), and Center for Epidemiological Depression Scale. Scales used to assess anxiety included: State Trait Anxiety Inventory, Kingston Caregiver Stress Scale, General Anxiety Disorder (GAD) scale, and Hamilton Anxiety Rating Scale. Scales used to assess stress included: Psychological Screening Inventory, Parenting Stress Index, Parental Stress Scale, and Perceived Stress Scale (PSS-10). Hospital Anxiety and Depression Scale (HADS) and General Health Questionnaire (GHQ) were used to measure both anxiety and depression across the studies, whilst Depression, Anxiety and Stress Scale (DASS-21) was used to measure depression, anxiety, and stress. Other studies employed scales developed by the researchers. The use of non-validated measures may be a limitation, and findings from these studies should be interpreted with appropriate caution.

### 3.3. Psychological Burden of Parents with Children with Neurodevelopmental Disorders

Overall, the studies included in this scoping review suggest that caregivers of children with neurodevelopmental disorders, including intellectual developmental disability, epilepsy, autism spectrum disorders, and cerebral palsy, or genetic disorders such as 22q11 Deletion Syndrome, experience considerable emotional and psychological burden, including higher levels of stress, anxiety, and depression. Reported prevalence rates of caregiver psychological distress varied substantially across studies, with the highest prevalence of 85.1% for depression and 85.8% for anxiety [34]. In contrast, lower rates have also been documented, including 8.27% of caregivers reporting very elevated depressive symptoms [9], and 5.2% of mothers scoring within the clinical range for depression [41].

A low prevalence of 6.0% anxiety was also noted [63]. A high proportion of caregivers, 94.6%, reported experiencing moderate to severe stress [21], whilst a lowest prevalence of 7.9% stress was reported specifically among fathers [63]. For the studies that utilized Patient Health Questionnaire-9 (PHQ-9) and Generalized Anxiety Disorder-7 (GAD-7) to respectively measure depression and anxiety, disparities were observed among the reported prevalences with a prevalence of 37.5% for depression and 43.3% for anxiety, [10] and 85.1% for depression and 85.8% for anxiety [34], whilst [32] reported a prevalence of major depressive disorder in respect to the DSM-5 as (25.45%).

### 3.4. Mental Health of Caregivers by Continent

Studies from Asian countries reported some of the highest prevalence rates of caregiver mental health issues. A recent study [21] reported that 94.6% of caregivers of children with neurodevelopmental disorders experienced moderate–severe stress, and 26% of mothers reported very high stress levels in another study [39]. The highest prevalence of anxiety (85.8%) and depression (85.1%) was also reported among parents of children with neurodevelopmental disorders [34]. The lowest prevalence for anxiety disorder was 18.33% [67], and 9.4% caregivers were reported to be severely depressed [46].

Studies from African countries reported consistently high prevalence of stress, anxiety, and depression, though more variable rates of caregiver distress were observed. For instance, 79% of caregivers were at risk of clinical depression, and 31.6% reported severe depression [57], with the lowest prevalence of 10.4% for depression [49]. Anxiety prevalence was also high, 56% was reported among caregivers of generalized epilepsy [26], whilst a low prevalence of 25.5% was recorded in another study [49]. The majority of participants (52.2%) in another study in South Africa reported very high clinically significant stress [22].

Studies from North America highlighted increased levels of stress and depression, but typically reported lower prevalence rates than in Africa or Asia. The prevalence of very elevated symptoms of depression among parents of children with developmental or learning disabilities was 8.27% [9], and 57% scored in the at-risk range for major depressive disorder [69]. High levels of stress were also reported among caregivers (45%) [52], and the prevalence of anxiety was 25% [38].

European studies also reported elevated levels of psychological distress among caregivers. A total of 43% of the caregivers (mothers) had total Parenting Stress Index (PSI) scores above the threshold for clinical assessment [55]. The majority of parents of children with intellectual disabilities were reported to meet the criteria for possible clinical depression and/or anxiety, with 63% for depression and 75% for anxiety [54]. The lowest reported prevalence of anxiety and depression among caregivers was respectively 8.1% and 6.8%. [71]. A total of 5.2% of mothers scored in the clinical range for depressive symptoms [41].

Oceania studies (Australia) contributed vital insight into paternal mental health, which is often underrepresented in caregiver research. The prevalence of symptoms of depression (7.9%), anxiety (6.0%), and stress (7.9%) was reported among fathers [63]. Other studies reported terminologies such as severe mental disturbance, poor mental health, and severe burden.

### 3.5. Correlates of Caregivers’ Psychological Issues

Factors associated with caregivers’ psychological issues such as stress, depression and anxiety include family history of psychiatric disorder, male gender of the child, age of the child, child’s behavior problems, daily stress from own needs and child’s care needs, co-morbidity in children, child diagnosis and severity of disability [10,28,47,52,56,61,63,64]. Higher stress, anxiety, and depression were also linked to support, including low social support, having no sources for psychological support, or lack of community support, lack of family support, or support by medical and educational professionals [27,28,29,65,68,70,71]. Sociodemographic factors associated with the risk of stress and other psychological issues include gender, unemployment, primary education, low socio-economic status, poor economic resources, marital status, and the age of the caregiver [23,50,57].

## 4. Discussion

Overall, the studies included in this scoping review suggest that caregivers of children with neurodevelopmental disorder experience a substantial and multifaceted psychological burden, shaped by both child-specific and systemic factors. This review highlights that behavioral difficulty, severity of disability, and comorbid conditions are consistently associated with elevated stress, anxiety, and depression, underscoring the intensive and ongoing nature of caregiving demands [10,41,48,50,66]. However, beyond these child-related characteristics, the findings point to the critical role of contextual stressors, including unmet personal needs, limited access to psychosocial support, and broader social protection gaps, suggesting that caregiver distress cannot be fully understood without considering structural influences [29,70]. Diverse measurement scales were used by different studies, making the effective comparison of prevalence estimates difficult. For instance, among the studies that utilized Patient Health Questionnaire-9 (PHQ-9) and Generalized Anxiety Disorder-7 (GAD-7) to respectively measure depression and anxiety, disparities were observed among the reported prevalences [10,34]. These lead to substantial heterogeneity in reported prevalence rates, with stress, anxiety, and depression reaching as high as 94.6%, 85.8%, and 85.1%, respectively. While these figures underscore the severity of caregiver burden, they also raise important methodological concerns about comparability and highlight the need for standardized assessment tools in future research. Variations in screening tools, diagnostic criteria, and study populations complicate cross-study comparisons and may lead to either an overestimation or an underestimation of the true prevalence. These findings augment the complexities in the literature regarding whether caregiver psychological outcomes are primarily driven by the child’s clinical profile or by modifiable environmental and policy-related factors. Addressing this burden, therefore, requires a dual approach: targeted interventions that support caregivers in managing child-related challenges, and systemic policies that enhance access to sustainable psychosocial resources and social support systems. Strengthening these areas may not only improve caregiver well-being but also lead to better developmental and health outcomes for children. These disparities in reported prevalence may also be attributed to methodological differences, diagnostic cut-off thresholds, and differences in study populations and settings, particularly where the studies were conducted, making effective comparison of prevalence rates challenging.

Anxiety and depression were significantly higher among caregivers, with a prevalence of 85.1% depression and 85.8% anxiety among caregivers of children with developmental disabilities [34]. These findings suggest that caregivers of children with developmental disorders are more prone to psychological distress. Though all caregivers experience comparatively increased psychological issues, mothers of children with neurodevelopmental disorders in particular were noted to experience (79.2%) a more pronounced level of stress compared to fathers [46]. Similarly, relatively more mothers, 94%, and 66.7% of fathers were also found to have either anxiety or depressive symptoms, or both [28]. This finding was also supported in other caregiver groups [72,73]. This may be attributed to the fact that mothers traditionally are the presumptive caregivers of children, while the societal norm is for fathers to provide financially for their families [74]. Additionally, women often have a natural bond with their children due to childbearing and are usually more closely and emotionally involved in caregiving, and are normally expected to be more involved with household work than men [75]. The plausible explanation is that this indirectly causes them to have more interaction with a child with a neurodevelopmental disorder, and the increased contact may have led to the observed heightened stress, anxiety, and depression. However, another study reported similar rates of anxiety and depression among both mothers and fathers [34], though the contrary has also been reported in another study [28]. Although prevalence rates of psychological issues among mothers were higher than for fathers [49], the articles that focused particularly on fathers in this scoping review highlight an important and often overlooked finding: fathers also experience significant levels of psychological distress, though they may be less likely to seek support or be included in research. Fathers’ work and daily stress from their own needs and children’s care needs appeared to be a significant predictor of their psychological issues [50,63], which can be explained by the societal role of fathers as financial providers.

The construct of parental “resolution” of the child’s diagnosis, particularly in situations where there are multiple diagnoses, may potentially play a role in the reported prevalence of stress, anxiety and depression among caregivers. Caregivers’ internal processing of the diagnosis interacts with parenting stress, anxiety, or depression to shape the parent–child relationship. Notably, resolution with the child’s diagnosis involves coming to terms with and accepting the diagnosis and its implications [76]. One study reported that parental resolution had a significant direct effect on the parent–child relationship, indicating that resolving the child’s diagnosis could potentially influence parents’ perceptions of their relationship with their child [77]. This resolution may also possibly lead to reduced stress, anxiety and depression among parents of caregivers with neurodevelopmental disorders.

In cases where the developmental disorder is due to a genetic disorder, such as 22q11 Deletion Syndrome (22q11DS), a high rate of psychiatric symptoms was reported, with 30% of caregivers meeting the criteria for symptoms of post-traumatic stress disorder (PTSD) [38]. Thus, aside from anxiety and depression, caregivers are at risk of PTSD symptoms over time. This highlights the distressing emotional impact experienced by caregivers, which may be compounded by the lack of knowledge on how to manage chronic and rare neurodevelopmental conditions, leading to the possibility of PTSD.

When disability is conceptualized in relation to the International Classification of Functioning, Disability and Health (ICF) framework, it may be understood not solely in terms of individual impairment or diagnostic condition, but also as resulting from the interaction between the person and environmental, relational, and social factors [78,79]. This highlights the importance of contextual factors, social policies, and support systems. The burden of care can be compounded in low-resource settings. In Africa, for instance, one study in Malawi reported that caregivers were unable to re-engage in income-generating activities due to caregiving demands, resulting in financial difficulties [65]. These experiences may be further influenced by a lack of social, emotional, and practical support from community and healthcare professionals, leading to feelings of isolation and emotional exhaustion [80]. Environmental factors, including access to respite services, financial resources, healthcare and community support, may mitigate caregiving-related stress, anxiety and depression [12,13]. Consistent with the International Classification of Functioning, Disability and Health (ICF) framework, psychological well-being can therefore be viewed as the dynamic interaction between individual functioning and personal factors/activities and caregiving demands, environmental facilitators or barriers, and the broader social and contextual circumstances in which caregiving occurs [78,79]. These findings indicate that caregiving for a child with neurodevelopmental disabilities is a common major mental health risk factor across cultures and countries, but may be particularly heightened where financial and social support is limited or non-existent.

Paternal and maternal age of caregivers are significantly associated with stress, anxiety, and depression [28,57,69], with high parental stress, for instance, linked with the advanced age of caregivers [22]. The psychological burdens increase with the age of caregivers, and the probable explanation is that caregivers have dealt with a child’s neurodevelopmental disorders over a long period of time, leading to cumulative emotional strain due to age-related factors, health challenges, increased caregiving demands, and reduced social support. One study noted that as the duration of caregiving increased, the caregiving burden became elevated, and caregivers’ anxiety and depression inadvertently worsened [72]. On the contrary, younger age has also been associated with increased psychological burden. This is also corroborated by another study, which reported that young adult caregivers reported significantly more symptoms of depression and anxiety [81]. These seemingly contradictory findings may reflect the multidimensional nature of caregiving burden across the lifespan. The relationship between caregiver age and psychological burden is likely non-linear and context-dependent, influenced by factors such as caregiving duration, socioeconomic resources, coping capacity, and availability of social support. Additionally, caregivers’ psychological difficulties can be understood not only as individual responses to caregiving demands but also as experiences shaped by the interaction of individual, relational, environmental, and contextual factors. Invariably, higher levels of psychological distress may reflect the cumulative effects of caregiving demands, perceived burden, limited coping resources, and uncertainty regarding the child’s condition and future needs.

### 4.1. Impact of the COVID-19 Pandemic

The COVID-19 pandemic exacerbated the already high mental health problems that caregivers of children with neurodevelopmental disabilities were experiencing and led to worsening psychological distress among caregivers. The most significant contributors to stress were closure of schools or therapeutic activities [7,13]. A significant proportion of caregivers felt stressed, with 68.2% feeling that their stress levels were higher compared to before the COVID-19 pandemic, and for those who screened positive for any of the psychological issues, a higher percentage was considered to have moderate or more severe symptoms [7]. The equal distribution of studies published up to 2019 and those from 2020 to 2025 (n = 28 in each period), on one hand, may suggest a sustained and consistent research interest in caregiver psychological well-being over time, rather than a recent surge confined to the COVID-19 era. On the other hand, the concentration of half of the included studies within a shorter, more recent timeframe could indicate an acceleration of research activity, potentially driven by heightened awareness of caregiver burden and the amplifying effects of pandemic-related stressors. Notably, between 2019 and 2021, corresponding to the early phase of the COVID-19 pandemic, a total of 12 studies were published. However, while the numerical balance appears even, the underlying research dynamics between these periods may differ substantially and should be interpreted with caution.

### 4.2. Study Limitations

Only studies published in the English language were included in the scoping review, which may have excluded some relevant studies in other languages, impacting the interpretation of the findings. Again, the overall search strategy may have been biased towards health and sciences databases, and searching other bibliographic databases may have generated additional relevant studies. Additionally, the included articles utilized various screening tools and different diagnostic classifications to determine symptoms of stress, anxiety, and depression, leading to variations in prevalence estimates. Therefore, the finding may not be generalizable. The use of non-validated measures in some studies may be a limitation; thus, findings from these studies should be interpreted with appropriate caution. Furthermore, since this is a scoping review, no formal quality appraisal of the included studies was undertaken; consequently, all studies meeting the inclusion criteria were incorporated into the data synthesis regardless of methodological rigor. Finally, an important limitation is that the availability and adequacy of social, family, and professional support were not systematically assessed within the individual studies included in this review. Consequently, the extent to which differences in access to such support may explain variations in caregiver psychological burden across studies remains unclear and represents a vital gap for future research.

Notwithstanding these limitations, this scoping review offers good insight into the prevalence and correlates of stress, anxiety, and depression among caregivers of children with neurodevelopmental disabilities.

### 4.3. Implications for Policy and Future Directions

The high levels of psychological distress observed in this scoping review among caregivers of children with neurodevelopmental disabilities highlight an urgent need for caregivers’ mental health to be prioritized and recognized as a public health issue. Policies that are not only child-focused interventions but also explicitly incorporate routine mental health screening, early identification, and ongoing psychosocial support for caregivers should be implemented, particularly within the pediatric community. Health systems should also ensure that mental health services for caregivers are timely, affordable, accessible, and destigmatized, particularly in low-resource settings where the burden of care is compounded by financial hardship and limited social support.

The disproportionately high psychological issues reported among mothers point to the need for gender-responsive policies that acknowledge caregiving inequities while avoiding the marginalization of fathers. Family-centered policies may promote shared caregiving responsibilities through workplace flexibility and targeted outreach that actively engage men in caregiving research and support programs. At the same time, interventions should shape caregiving roles, ensuring that support strategies are sensitive to cultural norms and contextually appropriate to prevent reinforcing traditional gender expectations, but instead help develop inclusive caregiver roles.

Findings related to caregiver age, duration of caregiving, and developmental conditions emphasize the need for long-term, adaptive support models rather than short-term interventions. Policies should support lifespan-oriented caregiver services with consideration of the caregiver’s age. Specialized caregiver education and peer-support networks, counseling, and trauma-informed care are particularly important for caregivers managing rare or neurodevelopmental conditions, where limited clinical knowledge may intensify anxiety and distress.

Additionally, social protection and economic support policies are vital components in relation to caregiver psychological burden. Evidence from low-resource settings demonstrates that caregiving demands often limit caregivers’ ability to engage in income-generating activities, thereby increasing stress, anxiety, and social isolation. Policymakers should therefore consider financial assistance, caregiver allowances, subsidized childcare, and respite care services as integral mental health interventions. In Africa, for instance, strengthening community-based support systems, including caregiver support groups and partnerships with non-governmental and faith-based organizations, may also enhance social connectedness and reduce emotional exhaustion.

Finally, the exacerbation of caregiver distress during the COVID-19 pandemic highlights the vulnerability of this population during crises and the need for resilient service delivery models. Future policy should prioritize continuity of educational, therapeutic, and respite services during public health emergencies through telehealth, community-based alternatives, and emergency social protection measures.

Despite the growing body of literature on caregiver psychological burden, several vital research gaps remain. There is a lack of longitudinal studies examining the long-term effects of stress, anxiety, and depression over the caregiving duration, limiting understanding of the long-term mental health trajectories. Considerable heterogeneity in study design, measurement tools, and diagnostic criteria also impedes meaningful comparison across studies and regions, underscoring the need for standardized, validated assessment instruments. Fathers, older caregivers, and caregivers in low- and middle-income countries remain underrepresented. Addressing these gaps through inclusive, methodologically rigorous, and intervention-focused research is essential to inform evidence-based policies and practices that adequately support caregivers of children with neurodevelopmental disabilities.

Future studies should prioritize longitudinal studies, standardized measurement tools, and include more fathers who are usually underrepresented caregiver groups, and expand evidence from low- and middle-income countries. Strengthening the evidence base in these areas will support the development of equitable, scalable, and sustainable policies that protect the mental health of caregivers across diverse contexts.

## 5. Conclusions

This scoping review demonstrates that caring for children with neurodevelopmental disabilities is consistently associated with high levels of stress, anxiety, and depression across diverse cultural and socioeconomic contexts. Despite methodological variations among studies, the evidence shows that caregivers, in particular mothers, but also fathers, constitute a vulnerable population whose mental health needs remain inadequately addressed. The burden is further intensified by factors such as prolonged caregiving duration, caregiver age, low-resource environments, lack of social support, and disasters such as the COVID-19 pandemic. Addressing these challenges requires coordinated, family-centered policies that integrate caregiver mental wellbeing into healthcare, social, and economic systems. It is also vital to strengthen research, standardize assessment tools, and expand access to integrated care and support. Expanding access to sustainable psychosocial and social protection interventions is a vital step toward improving caregiver well-being. This will ultimately have an indirect improvement in outcomes for children with neurodevelopmental disabilities.

## Data Availability

No new data were created or analyzed in this study. Data sharing is not applicable to this article.

## Supplementary Materials

The following supporting information can be downloaded at: https://www.mdpi.com/article/10.3390/brainsci16090982/s1. Ref. [16] is cited in the Supplementary Materials.

## Appendix A. Electronic Search Strategy for PubMed

**Search Number**	**Query**	**Sort By**	**Filters**	**Search Details**	**Results**
4	#1 AND #2 AND #3			(“Father”[Title/Abstract] OR “mother”[Title/Abstract] OR “caregiver*”[Title/Abstract] OR “parent*”[Title/Abstract] OR “guardian*”[Title/Abstract]) AND (“child* intellectual disability”[Title/Abstract] OR (“child*intellectual”[All Fields] AND “developmental disability”[Title/Abstract]) OR “child* developmental delay”[Title/Abstract] OR “child* neurodevelopmental disorder”[Title/Abstract] OR “child* learning disability”[Title/Abstract] OR “child* autism”[Title/Abstract] OR “child* autism spectrum disorder”[Title/Abstract] OR (“child*”[All Fields] AND “down syndrome”[Title/Abstract]) OR “child* cerebral palsy”[Title/Abstract] OR “adolescen* intellectual disability”[Title/Abstract] OR (“adolescen*”[All Fields] AND “intellectual developmental disability”[Title/Abstract]) OR (“adolescen*”[All Fields] AND “developmental delay”[Title/Abstract]) OR (“adolescen*”[All Fields] AND “neurodevelopmental disorder”[Title/Abstract]) OR “adolescen* learning disability”[Title/Abstract] OR “adolescen* autism”[Title/Abstract] OR “child* autism spectrum disorder”[Title/Abstract] OR (“adolescen*”[All Fields] AND “down syndrome”[Title/Abstract]) OR “adolescen* cerebral palsy”[Title/Abstract]) AND (“mental health”[Title/Abstract] OR “depression”[Title/Abstract] OR “anxiety”[Title/Abstract] OR “stress”[Title/Abstract] OR “psychological distress”[Title/Abstract] OR “mental health issues”[Title/Abstract] OR “major depressive disorder”[Title/Abstract] OR “anxiety disorder”[Title/Abstract] OR “generalized anxiety disorder”[Title/Abstract])	529
3	mental health[Title/Abstract] OR depression[Title/Abstract] OR anxiety[Title/Abstract] OR stress[Title/Abstract] OR psychological distress[Title/Abstract] OR mental health issues[Title/Abstract] OR major depressive disorder[Title/Abstract] OR anxiety disorder[Title/Abstract] OR generalized anxiety disorder[Title/Abstract]			“mental health”[Title/Abstract] OR “depression”[Title/Abstract] OR “anxiety”[Title/Abstract] OR “stress”[Title/Abstract] OR “psychological distress”[Title/Abstract] OR “mental health issues”[Title/Abstract] OR “major depressive disorder”[Title/Abstract] OR “anxiety disorder”[Title/Abstract] OR “generalized anxiety disorder”[Title/Abstract]	2,089,417
2	Child* intellectual disability[Title/Abstract] OR child*intellectual developmental disability[Title/Abstract] OR child* developmental delay[Title/Abstract] OR child* neurodevelopmental disorder[Title/Abstract] OR Child* Learning disability[Title/Abstract] OR Child* autism[Title/Abstract] OR child* autism spectrum disorder[Title/Abstract] OR Child* Down syndrome[Title/Abstract] OR Child* cerebral palsy[Title/Abstract] OR Adolescen* intellectual disability[Title/Abstract] OR Adolescen* intellectual developmental disability[Title/Abstract] OR Adolescen* developmental delay[Title/Abstract] OR Adolescen* neurodevelopmental disorder[Title/Abstract] OR Adolescen* Learning disability[Title/Abstract] OR Adolescen* autism[Title/Abstract] OR child* autism spectrum disorder[Title/Abstract] OR Adolescen* Down syndrome[Title/Abstract] OR Adolescen* cerebral palsy[Title/Abstract]			“child* intellectual disability”[Title/Abstract] OR (“child*intellectual”[All Fields] AND “developmental disability”[Title/Abstract]) OR “child* developmental delay”[Title/Abstract] OR “child* neurodevelopmental disorder”[Title/Abstract] OR “child* learning disability”[Title/Abstract] OR “child* autism”[Title/Abstract] OR “child* autism spectrum disorder”[Title/Abstract] OR (“child*”[All Fields] AND “down syndrome”[Title/Abstract]) OR “child* cerebral palsy”[Title/Abstract] OR “adolescen* intellectual disability”[Title/Abstract] OR (“adolescen*”[All Fields] AND “intellectual developmental disability”[Title/Abstract]) OR (“adolescen*”[All Fields] AND “developmental delay”[Title/Abstract]) OR (“adolescen*”[All Fields] AND “neurodevelopmental disorder”[Title/Abstract]) OR “adolescen* learning disability”[Title/Abstract] OR “adolescen* autism”[Title/Abstract] OR “child* autism spectrum disorder”[Title/Abstract] OR (“adolescen*”[All Fields] AND “down syndrome”[Title/Abstract]) OR “adolescen* cerebral palsy”[Title/Abstract]	18,618
1	Father[Title/Abstract] OR mother[Title/Abstract] OR caregiver*[Title/Abstract] OR parent*[Title/Abstract] OR guardian*[Title/Abstract]			“Father”[Title/Abstract] OR “mother”[Title/Abstract] OR “caregiver*”[Title/Abstract] OR “parent*”[Title/Abstract] OR “guardian*”[Title/Abstract]	843,383
#: Number.					
